# Standardized Scoring Tool and Weaning Guideline to Reduce Opioids in Critically Ill Neonates

**DOI:** 10.1097/pq9.0000000000000562

**Published:** 2022-06-14

**Authors:** Dipen Vyas, Vilmaris Quinones Cardona, Amanda Carroll, Catherine Markel, Megan Young, Rachel Fleishman

**Affiliations:** From the * Division of Neonatology, St. Christopher’s Hospital for Children, Philadelphia, Pennsylvania; † Department of Pediatrics, Drexel University College of Medicine, Philadelphia, Pennsylvania; ‡ Department of Nursing, St. Christopher’s Hospital for Children, Philadelphia, Pennsylvania; § Department of Pharmacy, St. Christopher’s Hospital for Children, Philadelphia, Pennsylvania; ¶ Division of Neonatology, Department of Pediatrics, Einstein Medical Center Philadelphia, Philadelphia, Pennsylvania.

## Abstract

**Introduction:**

Pain impacts brain development for neonates, causing deleterious neurodevelopmental outcomes. Prescription opioids for analgesia or sedation are common; however, prolonged opioid exposure in neonates is associated with neurodevelopmental impairment. Balancing the impact of inadequate pain control against prolonged opioid exposure in neonates is a clinical paradox. Therefore, we sought to decrease the average days of opioids used for analgesia or sedation in critically ill neonates at a level IV Neonatal Intensive Care Unit by 10% within 1 year.

**Methods:**

A multidisciplinary quality improvement team used the model for improvement, beginning with a Pareto analysis, and identified a lack of consistent approach to weaning opioids as a primary driver for prolonged exposure. The team utilized 2 main interventions: (1) a standardized withdrawal assessment tool-1 and (2) a risk-stratified opioid weaning guideline.

**Results:**

We demonstrated a reduction in mean opioid duration from 34.3 to 14.1 days, an increase in nursing withdrawal assessment tool-1 documentation from 20% to 90%, and an increase in the documented rationale for daily opioid dose in provider notes from 20% to 70%. Benzodiazepine use did not change.

**Conclusion:**

Standardized withdrawal assessments combined with risk-stratified weaning guidelines can decrease opioid use in critically ill neonates.

## INTRODUCTION

Pain impacts brain development for neonates, causing deleterious neurodevelopmental outcomes. It alters brain microstructural development in subcortical gray and white matter,^1^ reduces cortical thickness throughout the brain,^2^ slows the corticospinal tract development,^3^ and alters functional brain activity.^4^ These impacts extend into childhood. Pain is associated with altered cognitive and motor development and a higher prevalence of internalizing behaviors such as depression and anxiety later in life.^5–7^

Increased awareness of neonatal pain and its associated long-term outcomes has led to the prescription of opioids to alleviate pain in neonates.^8^ However, opioid exposure is not without impact on the developing brain. Routine use of morphine infusion in mechanically ventilated neonates is associated with lower overall intelligence quotient scores when compared with placebo.^9,10^ In addition, opioid exposure is associated with necrotizing enterocolitis, severe bronchopulmonary dysplasia, longer stay, and lower cognitive, motor, and language development scores at 2 years of age.^11^ Opioids may result in a longer duration of mechanical ventilation, hypotension, iatrogenic withdrawal, urinary retention, and decreased intestinal motility.^9,12,13^

Balancing the impact of inadequate pain control against prolonged opioid exposure in neonates is a clinical paradox. Wide practice variation exists in the use of opioids and sedatives between hospitals.^14–16^ How to best minimize pain and opioid exposure is unclear. Some units rely on protocols to prevent opioid initiation.^17,18^ Others emphasize the importance of treating pain and use management guidelines that center around measures of infant discomfort. Prior quality improvement studies in a non-Neonatal Intensive Care Unit (NICU) population achieved a 20% reduction in opioid use through standardized withdrawal assessment and weaning of opioids.^19,20^

Our goal was to limit the impact of both pain and opioids on the developing brain whereas not interfering with clinician autonomy to care for critically ill neonates at the height of their illness. With this in mind, we aimed to decrease the duration of opioid use for analgesia or sedation in critically ill neonates in our outborn level IV NICU by 10% within 1 year of project initiation.

## METHODS

### Context

St. Christopher’s Hospital for Children’s NICU is a 39-bed level 4 regional perinatal center in Philadelphia, PA, with 200−250 annual outborn admissions. Our unit provides the highest level of neonatal intensive care for those requiring advanced surgical services, therapeutic hypothermia, extracorporeal membrane oxygenation, and pediatric subspecialty services.

Inclusion criteria for this initiative were all neonates admitted to our NICU with a prescription for opioids for at least 1 day and survived until discharge. Exclusion criteria included prescription opioids for antenatal opioid exposure, transfer to different hospitals while on therapy, or death.

### Interventions

**Fig. 1.**
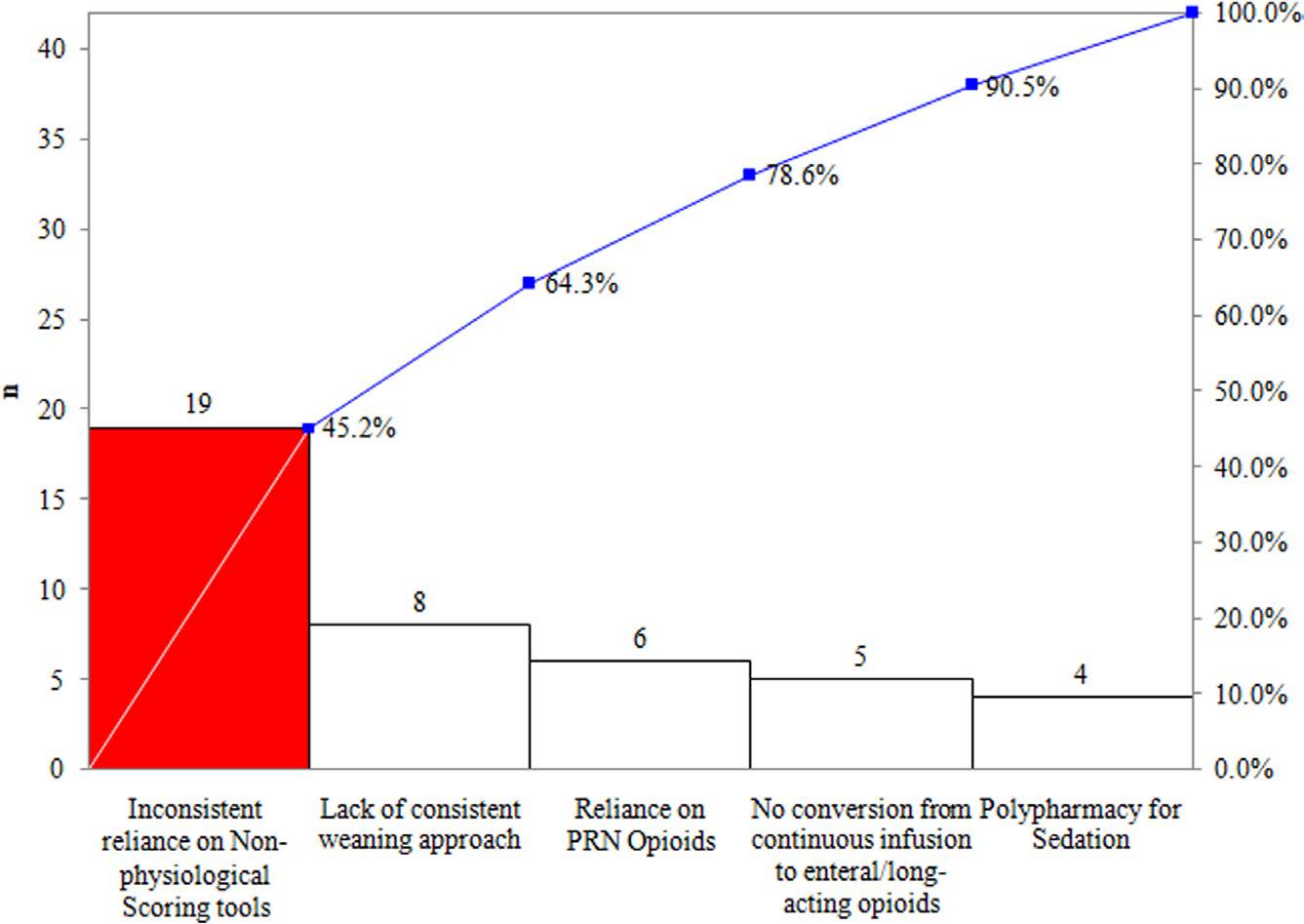
Pareto chart of primary drivers causing unnecessary prolonged opioid exposure.

We created a multidisciplinary quality improvement team involving neonatologists, a clinical pharmacist, nursing educators, and a bedside nurse in September 2019. The team used the Institute for Healthcare Model for Improvement, beginning with a Pareto analysis (Fig. 1). We identified a lack of standardized withdrawal assessment for opioids and an inconsistent approach to weaning as the primary drivers for prolonged exposure. Therefore, the team devised guidelines to improve consistency in management by facilitating decision-making with 2 key interventions: a standardized withdrawal assessment tool-1 (WAT-1) and a risk-stratified weaning guideline incorporating the WAT-1 scoring tool.

Before initiating this project, our NICU lacked a consistent approach to incorporating nursing assessment, physical exam, and withdrawal scores. Assessing the degree of iatrogenic opioid withdrawal on a given day and determining when and how to wean opioids was haphazard and informal. We used Finnegan scores for these patients. Therefore, to address the use of a scoring tool not intended for this population, we focused our efforts from September to December 2019 on educating providers and nursing staff on the WAT-1 scoring tool. We utilized preexisting division educational conferences to discuss iatrogenic opioid withdrawal, appropriate withdrawal assessment, and the rationale for WAT-1 scores as a tool. We did one-on-one sessions educating providers on the appropriate use of the weaning guideline before implementation. Also, nursing educators performed several educational sessions with nursing staff for both day and night shifts to ensure nurses monitored WAT-1 scoring on every neonate at risk of iatrogenic opioid withdrawal.

After 4 months of unit-wide education, we standardized clinical decision-making based on WAT-1 scores in January 2020. Any neonate receiving opioids had a withdrawal assessment score by nursing staff every 12 hours at the end of their shifts. The electronic health record (EHR) prompted nurses to complete WAT-1 scoring. Scores <3 were considered appropriate, and a score of 3 or higher meant withdrawal symptoms. Nurses used nonpharmacological or pharmacological interventions at their discretion and monitored repeat scores within 1 hour of the previous score to assess change. We monitored WAT-1 scores until infants were off medications for 72 hours and demonstrated no withdrawal signs. In addition to withdrawal assessments, we continued assessing pain scores using the Neonatal Pain, Agitation, and Sedation scale according to Joint Commission’s standards. Clinicians and nurses would prioritize pain control over weaning if neonates were in pain.

**Fig. 2.**
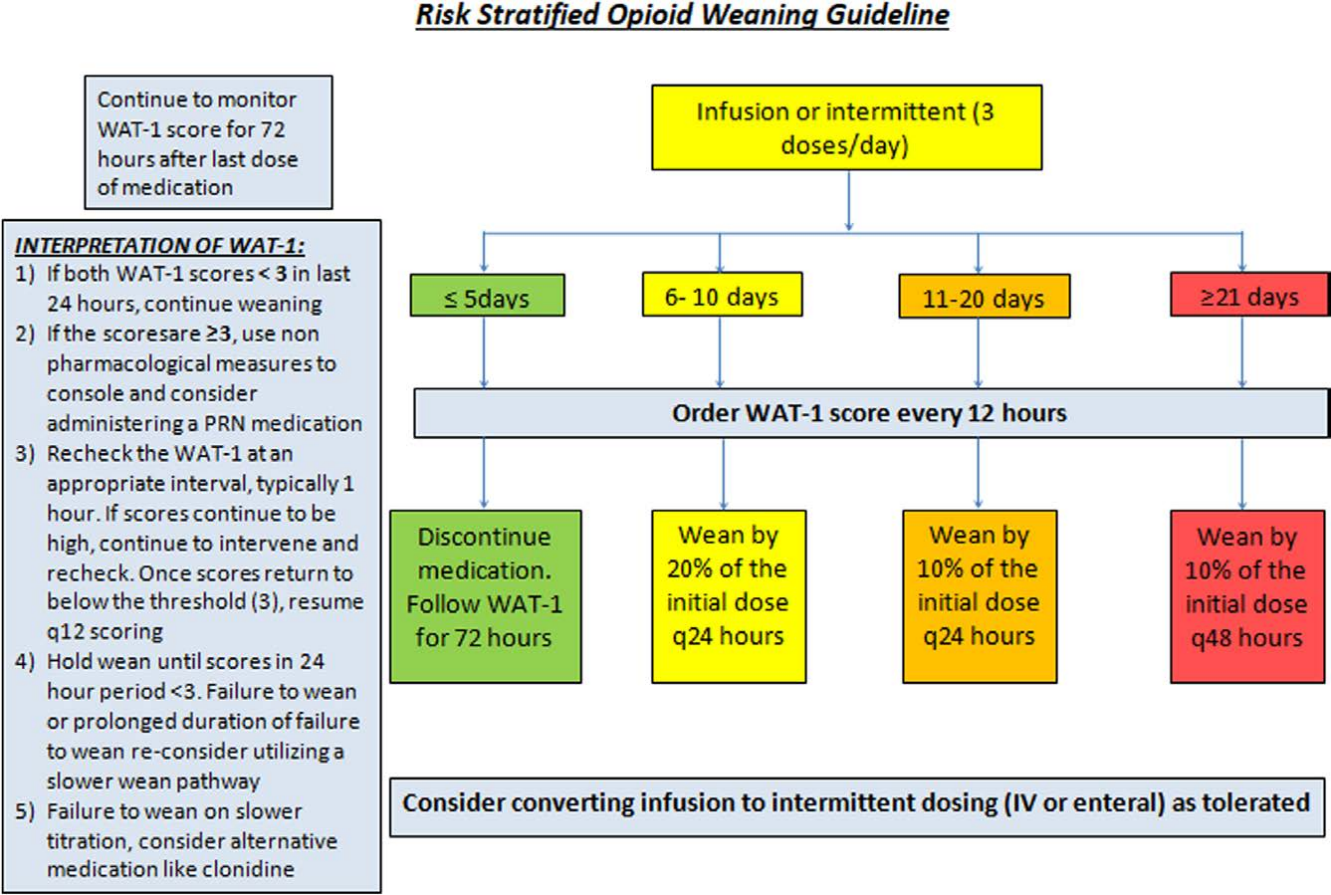
Risk-stratified opioid weaning guideline.

Our second intervention, concurrently implemented in January 2020, was a risk-stratified opioid weaning guideline. The guideline integrated WAT-1 scores into clinical decision-making. This guideline incorporated literature on weaning opioids and the consensus of our multidisciplinary quality improvement team with input from the entire physician and nursing staff. We suggested a weaning schedule based on the initial duration of exposure to opioid medications. For opioid duration younger than 5 days, no wean was recommended. For 6−10 days, 20% wean of the initial dose daily; for 11−20 days, 10% wean daily, and for longer than 21 days, wean by 10% every other day (Fig. 2).

To reinforce WAT-1 scoring and the guideline, we conducted reeducation between February and June 2020 during conferences and group discussions during rounds. A dedicated clinical pharmacist, present for daily rounds, reinforced guideline usage in real-time. Modifications to the note templates residents and midlevel providers used for daily rounds and sign-out prompted consideration of WAT-1 scoring and the duration of opioids. The team incorporated guidelines into the NICU clinical practice manual and displayed them on boards in the provider workrooms for easy access.

### Studying of Intervention

A single physician obtained data via a monthly retrospective review of EHRs during the baseline period (January 2019–December 2019) and intervention/sustainment period (January 2020–December 2020). Additionally, we deidentified data including gestational age, corrected gestation age, birth weight, indication for opioid prescription, duration of opioid therapy, WAT-1 score documentation by nursing, and discussions in daily progress notes regarding weaning of opioid therapy to compile the figures in this article.

### Measures

Our primary outcome measure was the average duration of total opioid therapy. The duration of opioid therapy for each patient was plotted on X-mR charts by the patient admission date. To review the effective use of the WAT-1 scoring tool, we used the percentage of nursing documentation of WAT-1 score every month as our process measure. We reviewed 5−10 randomly selected charts every month for neonates weaning from opioids to assess nursing documentation compliance. For assessment of discussions during rounds and weaning of opioids based on daily WAT-1 score and adherence to weaning guidelines, we followed documentation of reasoning for wean or lack thereof in 5−10 randomly selected daily progress notes every month. The most common concern among providers with reducing the duration of opioid therapy during the weaning period was the risk of increasing alternative sedation therapy. In our unit, the most used alternative therapy is benzodiazepine. Hence, we used the average duration of benzodiazepine therapy as our balancing measure and trended it as X-mR chart.

### Analysis

We used a statistical package for social sciences (IBM SPSS) Statistics for Windows software version 24 (Armonk, NY: IBM Corp) for descriptive analysis of gestational age, birth weight, and the indication of opioid use. We conducted pre/postgroup comparisons using an independent *t* test and chi-square test as appropriate. The outcome, process, and balancing measures were displayed using statistical process control X-mR charts and run charts. We used QI-Macros 2020 (Denver, CO: KnowWare International, Inc.) to analyze and generate statistical process control charts. Centerlines and 3-delta control limits were defined using standard approaches. We noted special cause variation when at least 8 consecutive points were above or below the centerline, 1 or more data points fell beyond the control limits, or 6 consecutive points trended in either direction.^21,22^ We adjusted centerlines based on detection of special cause signals.^23^ For run charts, we considered at least 6 consecutive data points above or below the median line as special cause variation and adjusted centerlines accordingly.

### Ethical Considerations

This local quality improvement QI project followed a local change in practice. Medical records were reviewed by a single physician already caring for the patients as part of the routine physician job description and did not compile personal health identifiers during the data collection for this project. As QI and not human subject research, the project did not require review and approval by the institutional review board.

## RESULTS

Between January 2019 to December 2020, there were 449 admissions to our NICU. Of these, 73 fulfilled the inclusion criteria (Figure 1, Supplemental Digital Content 1, http://links.lww.com/PQ9/A376). Table 1 shows the baseline characteristics of gestational age, corrected gestational age, birth weight, and the primary diagnosis for which these neonates received opioid therapy.

**Table 1.** Baseline Characteristics and Indications for Iatrogenic Opioid Use

	Baseline Mean (median)	Postintervention Mean (median)	*P*
Gestational age (weeks)	34.3 (36.5)	33.3 (35.5)	0.25
Corrected gestational age (weeks) on admission	35.6 (37.8)	34.9 (36.6)	0.29
Corrected gestation age (weeks) at initiation of opioids	37.6 (38.5)	37.4 (37.6)	0.45
Birth weight (g)	2,351 (2,565)	2,175 (2,556)	0.26
Duration of opioids (days)	N (%)	N (%)	0.61
≤5	12 (34.28)	18 (47.37)	
6–10	5 (14.28)	6 (15.79)	
11–21	9 (25.71)	8 (21.05)	
>21	9 (25.71)	6 (15.79)	
**Indication of opioid use**	**Baseline N (%)**	**Postintervention N (%)**	**0.01**
General surgery*	8 (22.86)	10 (26.31)	
HIE	7 (20.00)	3 (7.89)	
ECMO	5 (14.29)	4 (10.52)	
ENT/tracheostomy	4 (11.43)	1 (2.63)	
NEC/perforation	3 (8.57)	6 (15.79)	
BPD/PH	3 (8.57)	5 (13.16)	
MAS	2 (5.71)	1 (2.63)	
CT surgery	2 (5.71)	0 (0.00)	
PPHN	1 (2.86)	2 (5.26)	
Neurosurgery	0 (0.00)	6 (15.79)	
Total	35 (100)	38 (100)	

*Postoperative analgesia for all surgeries excluding post-ENT/ tracheostomy, CT surgery, and neurosurgery procedures.

BPD/PH, bronchopulmonary dysplasia with chronic pulmonary hypertension; CT surgery, cardiothoracic surgery; ECMO, extracorporeal membrane oxygenation; ENT, otolarngology; HIE, hypoxic ischemic encephalopathy; MAS, meconium aspiration syndrome; NEC, necrotizing enterocolitis; PPHN, persistent pulmonary hypertension in neonate.

**Fig. 3.**
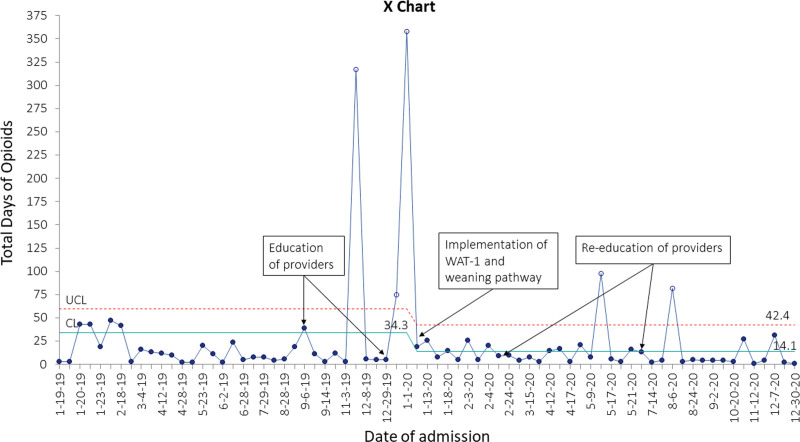
X chart for duration of opioid therapy for each patient in the study period based on the date of NICU admission.

After provider education, integration of the WAT-1 tool, and launching of the weaning guideline, we had a 59% reduction average duration of opioid therapy, from 34.3 to 14.1 days (Fig. 3) (Figure 2, Supplemental Digital Content 2, http://links.lww.com/PQ9/A376). There was special cause variation with 4 data points outside the upper control limits representing patients with bronchopulmonary dysplasia-associated pulmonary hypertension and opioid dependence. We excluded these data points from the centerline calculation as they did not represent the majority of opioid exposure in the unit. We separately analyzed data including these 4 data points, and the interpretation or timing of special cause variation did not change. With these 4 patients included, the average duration of opioid therapy for analgesia or sedation decreased from 31.7 to 13.6 days, a 57% reduction.

**Fig. 4.**
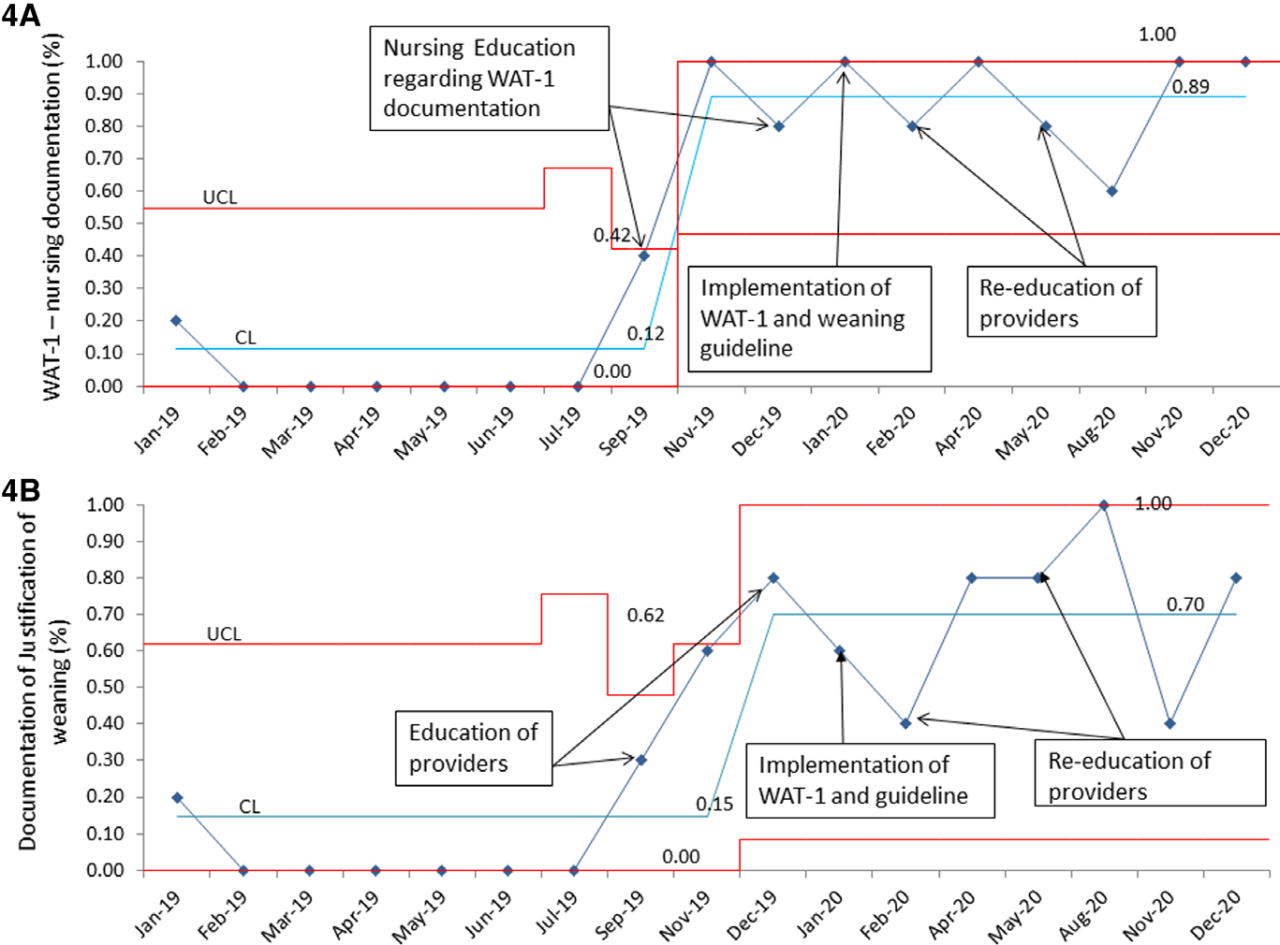
A, Run chart showing the monthly percentage of WAT-1 score documentation in EHR by the nursing staff. B, Run chart of the monthly percentage of documentation justifying opioid weaning in daily provider progress notes. EHR, electronic health record.

The monthly percentage of WAT-1 nursing documentation showed special cause variation with more than 6 consecutive data points above the median, a shift from 20% to 90% (Fig. 4A). In addition, the monthly percentage of documentation justifying opioid dose, an indirect measure to assess discussions during rounds for WAT-1 scores and weaning guidelines, also showed special cause variation with more than 6 data points above the median with a shift from 20% to 70% (Fig. 4B).

**Fig. 5.**
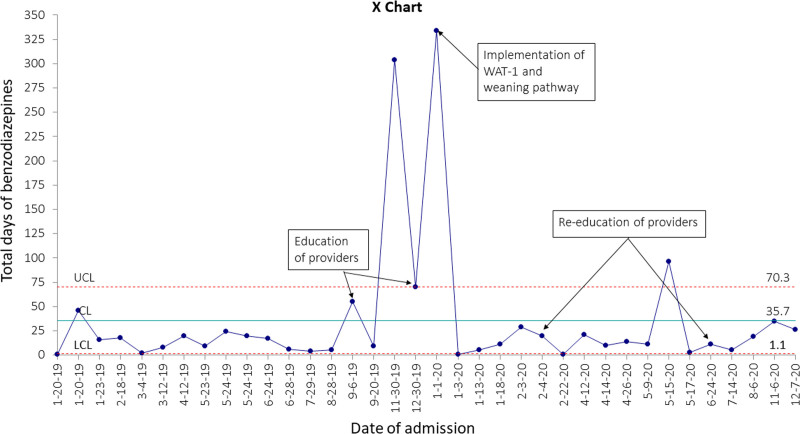
X chart of the total duration of benzodiazepine therapy per patient by NICU admission date.

The average duration of therapy for benzodiazepine, as a balancing measure, did not change (Fig. 5) (Figure 3, Supplemental Digital Content 3, http://links.lww.com/PQ9/A376). However, there was special cause variation with 3 data points above the upper control limit, reflecting the same patients in Figure 3 with bronchopulmonary dysplasia–associated pulmonary hypertension.

## DISCUSSION

By introducing a standardized withdrawal assessment scoring tool and design of a clinical guideline focusing on opioid weaning, we reduced the duration of opioid exposure for critically ill neonates in our Level IV NICU by 59%, exceeding our goal of 10%. This standardized scoring tool, the WAT-1 tool, was key to the success of this initiative.

In keeping with best-practice recommendations to base clinical decision-making about pain medications on validated assessment tools,^24^ we adopted the WAT-1. The WAT-1 tool was created and validated for ages 2 weeks to 18 years; however, it is used mainly in pediatric intensive care settings.^25,26^ Although there is limited WAT-1 literature among preterm neonates and in the NICU setting, we demonstrated successful use of WAT-1 scores in reducing the duration of opioid exposure in our population.

Before this project, our unit lacked a consistent approach to daily withdrawal assessment. Historically, we extrapolated from the modified Finnegan scoring for iatrogenic opioid exposure based on nursing and physician comfort although the modified Finnegan score was intended for otherwise healthy newborns with intrauterine drug exposure. Despite the convenience of using a familiar tool, signs like tachypnea and newborn-specific factors like feeding, sleep, nasal stuffiness, or Moro reflex are not relevant in those receiving opioids for critical illness on mechanical ventilation or postoperative analgesia. Because of the commonly prolonged stay in level IV NICUs, our population often reflects an older and more clinically complex group of infants than otherwise healthy newborns. Furthermore, in a recent study by Capino et al,^27^ a modified Finnegan score tended to overestimate withdrawal symptoms in postoperative cardiothoracic surgery infants with iatrogenic opioid abstinence syndrome, suggesting that this tool might not be reliable.

Although some neonatal studies reduced opioid prescription, most interventions developed guidelines for initiating pain medications or standardizing formal assessment tools for pain and sedation.^17,18,28,29^ Published neonatal studies have not focused on the weaning phase of opioids in the critically ill neonatal population. Given this gap in the literature, we accounted for the duration of opioid exposure and opted to standardize the weaning phase of these medications accordingly. In addition, adult, Pediatric, and Cardiac Intensive Care Unit literature focuses on the weaning phase of opioid therapy and emphasizes guidelines and WATs to standardize weaning and reduce opioid exposure.^19,20,30^ Based on these studies, we created an opioid weaning guideline for our level IV neonatal population. We were similarly able to show a marked reduction in the duration of opioid exposure in this NICU population through this approach.

Iatrogenic opioid withdrawal in neonates is of growing concern. In a large prospective cohort study, opioid prescription for sedation, or analgesia was as high as 26% of all ventilated neonates.^31^ Neonatal patients with iatrogenic opioid exposure, like older children, may develop opioid-induced hyperalgesia, tolerance, and withdrawal.^32^ Furthermore, the overall combination of total dose and consecutive days of opioid exposure directly correlates with the likelihood of withdrawal symptoms.^33^

Comfort management among critically ill neonates remains a challenge in the NICU. Despite the detrimental effects of poor pain management in neonates related to procedures and ventilation, medications used for such management are not without side effects. Therefore, we did not want to restrict providers from optimizing analgesia for perceived pain or when neonates were clinically labile. Instead, we focused our efforts on weaning and reducing the duration of opioids once the initial critical illness had resolved although reducing withdrawal symptoms through a standardized weaning phase.

Future interventions can incorporate nursing and pharmacist-driven weaning protocols, which improve compliance with weaning guidelines and reduce the duration of opioid therapy in the pediatric intensive care setting.^34,35^ Integrating the weaning guideline into the EHR is another intervention to improve sustainability.

## LIMITATIONS

This report is a single-center initiative focused on critically ill neonates with iatrogenic opioid exposure. Hence, the number of patients is small. However, as a referral center with only outborn neonates admitted to our NICU, we have a heterogeneous population of complex neonates requiring level 4 care. In addition to being a QI initiative, this fact explains differing indications for opioid use between the baseline versus postimplementation groups. However, despite the differences between groups, the interventions were uniform across various indications for opioid prescription and yielded positive results.

Additionally, a dedicated clinical pharmacist participating in rounds was integral to the success of this initiative and may not be readily available in every NICU. Finally, we focused on total days of opioid and benzodiazepine duration to ease data collection. Therefore, our data does not include daily dosage of morphine equivalent, duration of medication only during the weaning period, nor details of each sedative and analgesic prescription separately. We also did not use changes in pain scores as a balancing measure because incorporating clinical assessments of pain and withdrawal is nuanced and patient-dependent. Other units should consider the reproducibility of these interventions and tailor interventions related to the culture of opioid use for their patients.

At the time of this project, our institution’s ownership transitioned. Therefore, we anticipated a transition in the product and upkeep of the hospital’s EHR system. Electronic decision aids or new order sets to reinforce our project were not feasible but are natural next steps for this project.

## CONCLUSIONS

We reduced opioid duration among critically ill neonates in a level IV NICU using a dedicated multidisciplinary team focusing on a standardized weaning approach utilizing WAT-1 scores and risk-stratified opioid weaning guidelines once the critical period subsides.

## DISCLOSURE

The authors have no financial interest to declare in relation to the content of this article.

## ACKNOWLEDGMENTS

We want to acknowledge the valuable efforts of members of the neonatology team, including all the attendings, fellows, residents, nurse practitioners, and registered nurses at St Christopher Hospital for Children’s who assisted with this project.

## Supplementary Material


