# Supplementary figures and images for: Standardized Scoring Tool and Weaning Guideline to Reduce Opioids in Critically Ill Neonates
Source: Pediatr Qual Saf. 2022 Jun 14;7(3):e562. doi: 10.1097/pq9.0000000000000562 (PMC9197367; doi:10.1097/pq9.0000000000000562)

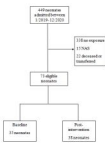

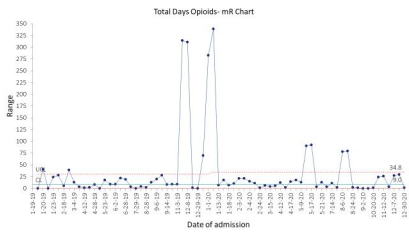

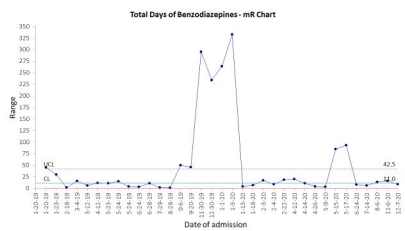

Supplement: Supplementary file 1 [file pqs-7-e562-s001.pdf]
